# Supplementary material for: Network-driven anomalous transport is a fundamental component of brain microvascular dysfunction
Source: Nat Commun. 2021 Dec 15;12:7295. doi: 10.1038/s41467-021-27534-8 (PMC8674232; doi:10.1038/s41467-021-27534-8)
Supplement: Supplementary file 1 — Supplementary Information [file 41467_2021_27534_MOESM1_ESM.pdf]

Supplementary Information for "Network-driven anomalous transport is a fundamental component of brain microvascular dysfunction"

Florian Goirand, Tanguy Le Borgne and Sylvie Lorthois

A. Blood flow simulations in intracortical networks

We use two large postmortem datasets ($\sim 15,000$ vessel segments in ~ 1 mm^3 regions) from the mouse vibrissa primary sensory (vS1) cortex of two mice, obtained by [1, 2]. One of them is displayed in Supplementary Figure S1**a-b** and Supplementary Movie S1, where vessel types (arteriole, capillary, venule) are outlined. We describe the vasculature as a network of interconnected tubes where arterioles and venules respectively act as inlets and outlets for blood flow. We compute the stationary flow rate distribution using a nonlinear network approach described in Pries et al. [3], Lorthois et al. [4] and Cruz-Hernández et al. [5] and implemented in a custom-built C++ code [6]. This approach, where blood is considered as a homogeneous fluid and red blood cells are treated as a volume fraction (haematocrit), accounts for the complex rheological properties of blood flow in microcirculation through two in vivo empirical laws. The first one describes the average dissipation at vessel scale through an apparent viscosity which depends on the tube diameter and haematocrit, so that a linear relationship between the flow rate and the pressure drop can be written in each vessel [7, 8]:

$$Q_{ij} = \frac{\pi d_{ij}^4}{128 \mu_{ij} l_{ij}} \Delta_{ij} P \tag{S1}$$

where Q_{ij}, d_{ij}, l_{ij}, $\Delta_{ij}P$ and μ_{ij} are respectively the flow rate, the mean diameter, the arc length, i.e. accounting for vessel tortuosity, the pressure difference and the apparent viscosity associated to vessel ij. The distribution of haematocrit in the network and phase-separation effects are captured by the second empirical law [9] that links haematocrit and flow rate ratios at diverging bifurcations.

This problem is nonlinear and is solved iteratively (see e.g. [4, 8, 10]) with the following boundary conditions: imposed physiological pressures at network inlets (one-connected arteriolar vertices located at the top-surface) and outlets (one-connected venular vertices located at the top-surface), where $P_A = 10640$ Pa and $P_V = 2660$ Pa, respectively, imposed haematocrit at network inlets (H=0.4), and no-flow at the bottom of the sample. Pseudo-periodic boundary conditions are used at network side faces as described in [5], with the additional constraints that, to connect two capillary vertices located on opposite side faces, their projections on a parallel plane are closer than 80 μm and that pressures in cut arterioles (resp. venules) are equal to the mean pressure of arterioles (resp. venules) of equal diameter at same depth in the network.

This yields the pressure, the flow rate and the haematocrit within the network, without any free parameter. The flow distribution in the network of Supplementary Figure S1**a-b** is displayed in Supplementary Figure S1**c-d**. Moreover, in Supplementary Figure S1**f**, we present

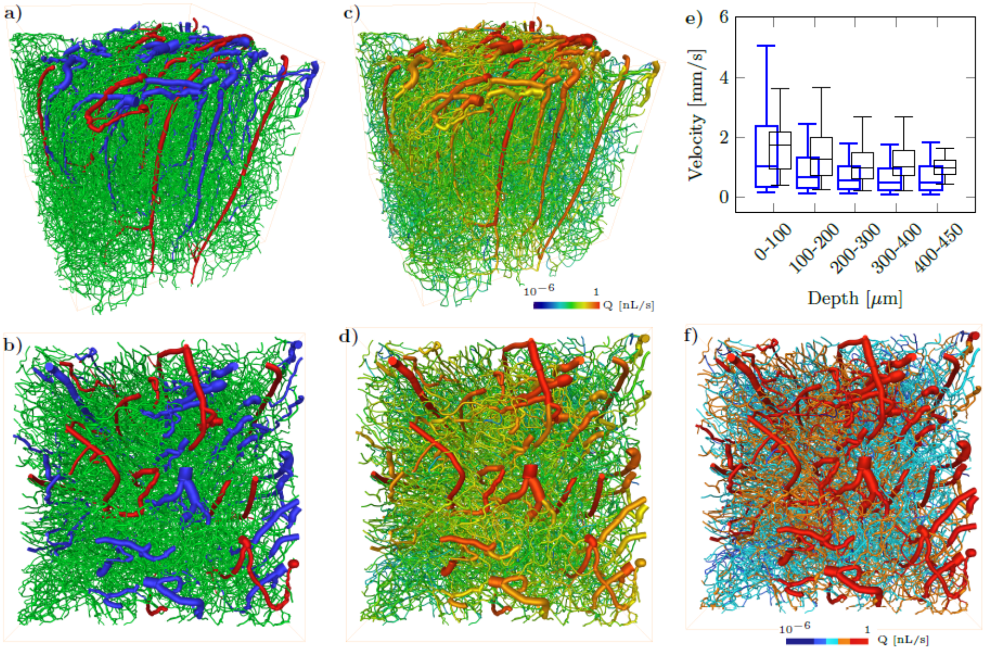

Supplementary Figure S1: 3D rendering of the microvascular network, showing vessel types and flow distributions. a-b) Snapshot of the first 1mm^3 mouse brain sample used to model the brain blood flow transport properties, side and top view (see Supplementary Movie S1), respectively. Arterioles are displayed in red, venules in blue and capillary vessels in green. Network inlets are defined as arteriolar vertices located at the top surface (pial surface) and connected only to a single other vertex, as apparent in b). Similarly, network outlets are defined as venular vertices located at the top surface and connected only to a single other vertex. **c-d)** Flow rate distribution using a continuous logarithmic color scale, side and top view respectively. **e)** Comparison of simulated capillary blood velocity statistics (1st decile, 1st quartile, median, 3rd quartile, 9th decile) with capillary red blood cell velocity statistics measured experimentally at different cortical depths within 100 micron-thick slices. Blue: simulated average blood velocity ($4Q/\pi d^2$) from the present study. The respective number of capillary vessels in each slice, from surface to depth, is 807, 1897, 1593, 1749 and 1150; Black: experimental red-blood cell velocity data extracted from [22]. **f)** Same as panel **d** using a discontinuous color scale as in Fig. 1**a** of the main manuscript with blue shades for $Q < Q_c$ and red shades for $Q > Q_c$.

the top view of Fig. 1 of the main manuscript, showing the flow distribution with a discontinuous scale, highlighting the different flow regimes. The above flow distributions have been validated by comparison to in vivo measurements in mouse (see Supplementary Figure 15g-h in Cruz-Hernández et al. [5]). In addition, the range of simulated velocities at different depths in the cortical microvessel network is consistent with experimental measurements (Supplementary Figure S1**e**). This supports the choice of the no-flow boundary condition at the bottom of the sample, which is the most uncertain one due to the lack of highly resolved in vivo flow data in the deep cortical layers.

For a given network architecture, the above linear system behaves linearly as a function of the perfusion pressure ($P_A - P_B$). The apparent viscosity in each vessel is indeed independent on the flow rate and, similarly, the parametrization of phase separation only depends on the flow ratio between the daughter branches. Thus, hypoperfusion resulting from a decreased perfusion pressure yields flow distributions which can be linearly deduced from the above simulations, for

which all parameters, including the perfusion pressure, correspond to physiological data. When a small proportion of capillary vessels is occluded, which slightly modifies the network architecture, all velocities remain, at first order, proportional to the mean flow rate.

B. Network exploration by Lagrangian trajectories and derived quantities

Blood transport is solved by particle tracking (see Materials and Methods, Fig. 2**a** and corresponding Supplementary Movie S2). Examples of Lagrangian trajectories originating from a given arteriole (highlighted by arrows in the figure) are displayed in Supplementary Figure S2**a-d**. Note that, because of the pseudo-periodic boundary conditions, these trajectories, can cross a side of the domain and connect to venules on the opposite side.

The proportion of vessels where intravascular diffusive transport dominates over advective transport (i.e. $Pe < 1$) for oxygen and amyloid-β in physiological conditions is estimated based on the vessel Péclet number $Pe = t/\tau_D$, where t is the local transit time and τ_D the diffusion time of the considered specie ($D_{O_2} = 2.10^{-9}$ m^2.s^{-1} and $D_{A\beta} = 6.10^{-11}$ m^2.s^{-1} [11]). For oxygen and amyloid-β in physiological conditions, it is respectively equal to 9% and 2%. Diffusion is taken into account in the Lagrangian statistics by replacing, for these vessels, the local (advective) transit time t by the local diffusion time ($t_D = l^2/D$).

The travel time distribution $p_{\mathcal{T}}(\mathcal{T})$ is computed from the Probability Density Function (PDF) of particle travel times from any inlet arteriole to any outlet venule. The fraction of travel times superior to a given threshold time $\mathcal{T}'$ is thus $f_p(\mathcal{T} > \mathcal{T}') = \int_{\mathcal{T}'}^{\infty} d\mathcal{T} p_{\mathcal{T}}(\mathcal{T})$. To evaluate how different pathways contribute to the transport dynamics within the network, we also quantified the fraction of vessels irrigated by trajectories of different travel times. We thus defined the fraction $f_v(\mathcal{T} > \mathcal{T}')$ of vessels in the network only visited by particles with travel times larger than $\mathcal{T}'$. The two fractions f_p and f_v are approximately equal, as shown in Supplementary Figure S2**e** from oxygen transport simulations, indicating that the statistics of travel times measured at the outlet are representative of the travel time statistics within the network.

To understand how particles of different lengths explore the different vessels (arterioles, venules, capillaries), we computed the average number of visited capillaries $\overline{n_c}$ as a function of trajectory length L (Supplementary Figure S2**f**). The number of visited capillaries initially increases until $L \approx 20$ and then reaches a plateau at $\overline{n_c} \approx 10$. Above L_c, the number of visited capillaries increases again linearly. Therefore below L_c, trajectories increases their lengths by visiting more arterioles and venules, as they penetrate deeper into the network (see Supplementary Figure S2**a-b** and schematic of Fig. 3 in the main manuscript). Above L_c, the steep increase of the number of visited capillaries with trajectory length reflects the exploration of the deep capillary bed by longer blood flow paths (see Supplementary Figure S2**c-d** and schematic of Fig. 3 in the main manuscript).

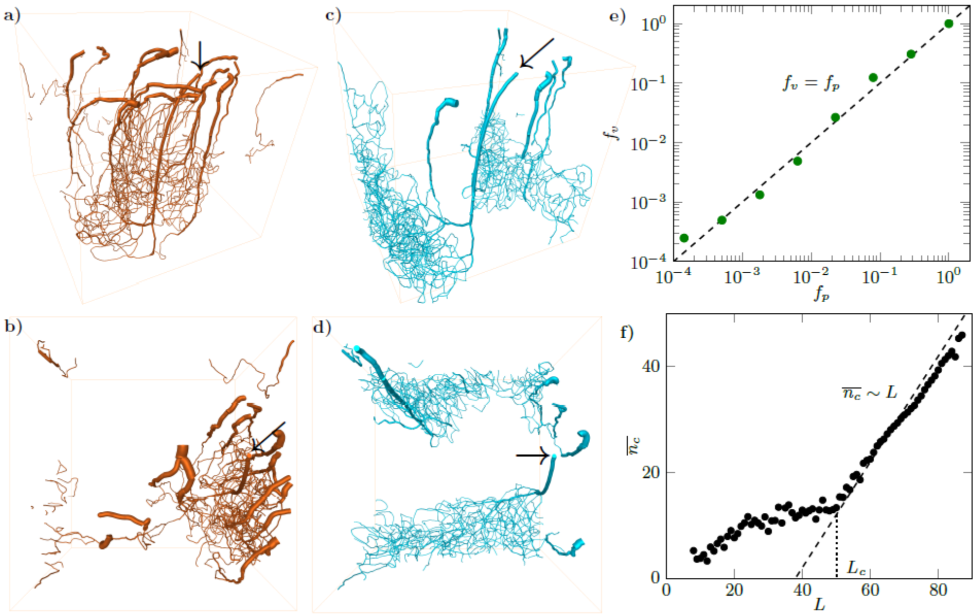

Supplementary Figure S2: Exploration of the network by Lagrangian trajectories a-b) Snapshots showing all particle trajectories originating from a single arteriole (highlighted by the arrow), with length smaller than $L = 30$, respectively side and top view and **c-d)** with length larger than $L = 70$. Note that, because of the pseudo-periodic boundary conditions, trajectories originating from a given arteriole, can cross a side of the domain and connect to venules on the opposite side. Trajectories are the same as in Fig. 1 of the main manuscript. **e)** Fraction $f_v(\mathcal{T} > \mathcal{T}')$ of vessels only visited by particles with travel times from the inlet to the vessel larger than $\mathcal{T}'$ as a function of the fraction $f_p(\mathcal{T} > \mathcal{T}')$ of network travel times larger than $\mathcal{T}'$. The dashed line represents $f_p = f_v$. **f)** Evolution of the average number of capillaries $\overline{n_c}$ within trajectories as a function of trajectory length L. The dashed line highlights the linear tendency $\overline{n_c} \sim L$.

C. Dipole flows on networks

To investigate the similarities and differences between the results obtained in intracortical networks and the results obtained in simpler networks, we compared the flow and transport statistics of different systems. We considered i) the classical solution of dipole flow in a 2D finite size continuous homogeneous medium [12] (Supplementary Figure S3**a**), ii) a dipole flow in a 2D square lattice with homogeneous conductances (Supplementary Figure S3**b**), and iii) a superposition of multiple dipole flows in a 3D random space-filling network with homogeneous conductances constructed following the method of Smith et al. [13] to reproduce the topological and functional properties of intracortical capillary networks (Supplementary Figure S3**c**). The boundary conditions are the following. For case i), the flow field is confined in a disk of radius R, so that the radial component of the velocity is zero on the boundary of the disk. For case ii), there is no interrupted segment at the periphery of the domain (top, bottom and lateral faces), so that no boundary condition has to be imposed there. For case iii), no flow boundary conditions are imposed on all interrupted segments at the periphery of the domain, except those randomly chosen as sources and sinks for dipolar injection at the top surface).

We first recall basic theoretical results derived by Kurowski et al. (1994) [12] for a dipole flow created by a source and sink separated by a distance a in a homogeneous layer of thickness

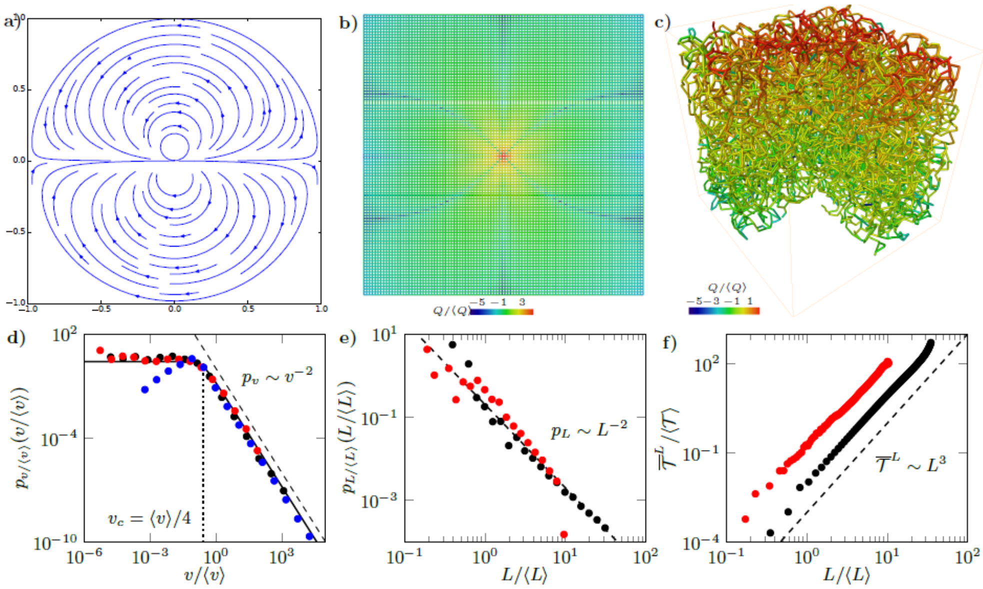

Supplementary Figure S3: Blood flow and trajectory statistics in dipole flows of increasing complexity. **a)** Streamlines for a single dipole flow in a 2D finite size continuous medium plotted using the solution of Kurowski et al. [12]. **b)** Flow distribution for a single dipole flow in a 2D square lattice with homogeneous conductances obtained by numerical simulation. The color scale represents the logarithm of $Q/\langle Q\rangle$. **c)** Flow distribution generated by a random distribution of inlet and outlet points on the upper surface of a synthetic 3D space-filling disordered network. The color scale represents the logarithm of $Q/\langle Q\rangle$. A quarter of the volume has been removed to enable visualization of the central part of the network. **d)** Velocity PDFs for the dipole flow in a finite continuous medium (blue), the dipole flow in a square lattice (black) and multiple dipole flows in a 3D random network (red), **e)** PDFs of trajectory lengths for the dipole flow in a square lattice (black) and multiple dipole flows in a 3D random network (red), and **f)** relationship between average travel time and trajectory length for the dipole flow in a square lattice (black) and multiple dipole flows in a 3D random network (red).

d limited by a circular impervious boundary of radius R. Taking the center of the line joining the source and sink as the reference point ($r = 0$), the velocity field can be expressed in polar coordinates as:

$$\begin{cases} v_r = \dfrac{q_0 a}{2\pi d}\cos\theta\left(\dfrac{1}{r^2}-\dfrac{1}{R^2}\right) \\ v_\theta = \dfrac{q_0 a}{2\pi d}\sin\theta\left(\dfrac{1}{r^2}+\dfrac{1}{R^2}\right) \end{cases} \tag{S2}$$

where r is the distance from the center and q_0 is the inlet and outlet flow rate. The velocity magnitude $v = \sqrt{v_r^2 + v_\theta^2}$ is,

$$v = \frac{q_0 a}{2\pi d}\sqrt{\cos\theta^2\left(\frac{1}{r^2}-\frac{1}{R^2}\right)^2 + \sin\theta^2\left(\frac{1}{r^2}+\frac{1}{R^2}\right)^2} \tag{S3}$$

For $r \ll R$, the velocity magnitude thus evolves as,

$$v(r) \underset{r\ll R}{\sim} \frac{q_0}{r^2}. \tag{S4}$$

which leads through the change of variable $p_v(v)dv = p_r(r)rdr$ to the velocity PDF,

$$p_v(v) \sim q_0 v^{-2} \tag{S5}$$

This scaling is verified in Supplementary Figure S3**d** for $v > v_c$, with the characteristic velocity $v_c = \langle v \rangle/4$. Below v_c, the PDF evolves as $p_v \sim v$. The large velocity scaling $p_v(v) \sim v^{-2}$ is also recovered for the dipole flow in a square lattice (Supplementary Figure S3**b**). However, the low velocity regime is replaced by a uniform distribution, as observed in our biological network. As discussed in the main manuscript, this confirms that the uniform distribution at low flows is related to the network structure.

Far from the dipole, the streamlines may be approximated as circles of radius r and length $L = 2\pi r$ [12]. The travel time derived by integration of the velocity along r given by Eq. (S4) is thus,

$$\overline{\mathcal{T}} = \int_0^L \frac{dr}{v(r)} \sim L^3 \tag{S6}$$

This is verified in Supplementary Figure S3**f** for a single dipole flow in a 2D square network (Supplementary Figure S3**b**). Kurowski et al. (1994) have further shown that the resulting travel time PDF for a single dipole in a homogeneous 2D medium is [12],

$$p_{\mathcal{T}}(\mathcal{T}) \sim \mathcal{T}^{-4/3} \exp(-\mathcal{T}/\mathcal{T}_c) \tag{S7}$$

where $\mathcal{T}_c = R^3/q_0$. Through the change of variable $p_L(L)dL = p_{\mathcal{T}}(\mathcal{T})d\mathcal{T}$ this leads to,

$$p_L(L) \sim L^{-2}, \tag{S8}$$

which is verified in Supplementary Figure S3**e** for a single dipole flow on a homogeneous 2D network.

We have investigated the statistical measurements described above for flow in 3D random space-filling network with homogeneous conductances (Supplementary Figure S3**c**), which are representative of the capillary bed structure [13]. Sixteen inlet points and 16 outlet points were randomly distributed at the surface of a random cubic network of $\sim 25^3$ vessels. Boundary conditions are imposed pressure on inlets and outlets and no flow on all other vessels at the boundary. The statistics of velocities and trajectory lengths, as well as the average travel time dependency with the trajectory length, appear to all follow the same statistics as the 2D network (Supplementary Figure S3**d-f**). This suggests that although the system is three-dimensional, the streamline patterns behave statistically as 2D dipoles. This behavior results from the multiple dipolar injection and extraction at the surface of the network, which constrains the streamlines to extend mostly vertically in the network, therefore being topologically equivalent to 2D dipoles.

In 3D systems with embedded dipoles far from each other, the velocity is expected to decay as $v(r) \sim r^{-3}$, leading from Eq. (S6) to the mean travel time:

$$\overline{\mathcal{T}} \sim L^4. \tag{S9}$$

This scaling is observed for large trajectory lengths in our microvascular network simulations (see inset of Fig. 2**c** in the main document and Supplementary Figure S4**b**). We interpret this transition to 3D dipole flows as resulting from the decrease in density of arterioles and venules in the depth of the cortex [14]. At their capillary ends, deep arterioles and venules are further from each other compared to their typical separation distance at the surface and the streamline patterns are therefore less constrained laterally and can develop large trajectories in the capillary bed (see schematic of Fig. 3 in the main manuscript), following the characteristic scaling of 3D dipoles.

As discussed above, many characteristics of dipole flow in continuous systems are recovered in the periodic lattices and random networks considered above. A notable difference concerns the flow distribution in the low flow range: the flow rate PDF for both periodic and random networks exhibits a plateau at low values, a signature of the network structure which is consistent with the findings of Alim et al. [15]. The latter suggest that flow distributions in random networks follow exponential PDFs, leading to the plateau at small flows. This uniform distribution of flow at low values, also observed in our biological networks, is at the origin of the broad distribution of vessel transit time discussed in the main manuscript. Thus, in the large flow rate and short trajectory length regime, microvascular networks are analogous to dipole flows in that they follow the scalings of Eq. (S5) and Eq. (S8) (see Fig. 1**b** and Fig. 2**b** in the main manuscript). However, the relationship between travel times and trajectory lengths (see Inset of Fig. 2c in the main text) is different from that expected for simple dipole flow (Eq. (S6)). The later exhibits a linear tendency for $L < L_c$ and then a power law with exponent 4, similar to Eq. (S9). We understand this difference as follows.

Trajectory lengths smaller than L_c, i.e in the first linear regime, correspond to trajectories with direct connections occurring at different depths between neighboring arterioles and venules [16], see schematic of Fig. 3 and Supplementary Figure S2**a**,**b**, where most venules connected to the injection arteriole lie in a cylindrical region of radius $\sim 700\ \mu m$ around it. Depending on the trajectory length L, these connections occur at different depths, but the pressure drop throughout the capillary bed is approximately constant. In fact, because they have larger diameters, pressure drops within arterioles and venules are small compared to pressure drops throughout the capillary network. Consistently, the number of visited capillaries in these trajectories depends weakly on the trajectory length (Supplementary Figure S2**f**). This implies that the average capillary transit time in these trajectories is the same for any $L < L_c$. Hence, as the trajectory length increases, the additional visited vessels belong to arterioles and venules for $L < L_c$. As discussed in the main mansucript, the linear scaling of $\overline{\mathcal{T}}^L(L)$ indicates that the average transit time t in such vessels remains approximately constant as trajectories explore deeper sections of the network.

Trajectories of lengths larger than L_c correspond to connections between arterioles and more distant venules (Fig. 3 and Supplementary Figure S2**c**,**d**), and include a number of capillaries linearly growing with the trajectory length (Supplementary Figure S2**f**). As explained above, in the deep part of the cortex, blood trajectories are driven by the 3D dipolar nature of the flow in between these distant arteriole and venules. Therefore, they follow the average time-length scaling expected for 3D dipole flows (Eq. S9).

D. Mean field transport model

The mean field transport model $p_{\overline{\mathcal{T}}}(\overline{\mathcal{T}})$ is derived by associating to each trajectory of length L a mean travel time, i.e. averaged over all trajectories of same length. This is equivalent to removing the noise induced by flow fluctuations linked to the random network structure. The mean field model is obtained analytically from the average travel time $\overline{\mathcal{T}}^L$ for a given trajectory length L and the trajectory length PDF $p_L(L)$ using the change of variable $p_{\overline{\mathcal{T}}}(\overline{\mathcal{T}})d\overline{\mathcal{T}} = p_L(L)dL$. The average travel time is characterized by a transition from a linear to power law behavior, driven by the flow organization respectively in the superficial and deep microvascular network (Eq. (5)

in the main manuscript and Appendix **C**),

$$\begin{cases} \overline{\mathcal{T}}^L(L) \approx \tau_1(L - L_0) + \mathcal{T}_0 & L_0 < L \leq L_c \\ \overline{\mathcal{T}}^L(L) \approx \mathcal{T}_c \left(\frac{L}{L_c}\right)^4 & L > L_c \end{cases} \tag{S10}$$

These trends are shown in linear and loglog plots in Supplementary Figure S4**a-b** (same as inset of Fig. 2 **c** in the main manuscript). The trajectory length PDF (Eq. (4) and Fig. 2**b** of the main manuscript) is

$$\begin{cases} p_L(L) \sim L^{-2} & L_0 < L \leq L_c \\ p_L(L) \sim \exp(-L/L^*) & L > L_c \end{cases} \tag{S11}$$

The mean field travel time PDF is $p_{\overline{\mathcal{T}}} = p_L(L(\overline{\mathcal{T}}))\frac{dL}{d\overline{\mathcal{T}}}$, leading to a transition from a power law to a stretched exponential:

$$\begin{cases} p_{\overline{\mathcal{T}}}(\overline{\mathcal{T}}) \sim \left((\overline{\mathcal{T}} - \mathcal{T}_0)/\tau_1 + L_0\right)^{-2}, & \text{for } \overline{\mathcal{T}} < \mathcal{T}_c \\ p_{\overline{\mathcal{T}}}(\overline{\mathcal{T}}) \sim \left(\frac{\overline{\mathcal{T}}}{\mathcal{T}_c}\right)^{-3/4} \exp\left(-\frac{L_c}{L^*}\left(\frac{\overline{\mathcal{T}}}{\mathcal{T}_c}\right)^{1/4}\right), & \text{for } \overline{\mathcal{T}} > \mathcal{T}_c \end{cases} \tag{S12}$$

We compare this analytical prediction to the numerical estimation of the mean field travel time PDF $p_{\overline{\mathcal{T}}}(\overline{\mathcal{T}})$, obtained by computing $\overline{\mathcal{T}}^L$ for each trajectory length and calculating its PDF for all trajectory lengths (Supplementary Figure S4**c**). The mean field travel time PDF is identifcal to the full travel time PDF up to a time of about $\mathcal{T} = 10$ s. Above this time, the full travel time PDF deviates to follow the power law trend $p_{\mathcal{T}} \sim \mathcal{T}^{-3}$, resulting from the noise component (see Appendix **E** and **F**). The analytical prediction of Eq. (S12) is in good agreement with the computed mean field PDF over the full range of times (Supplementary Figure S4**c**).

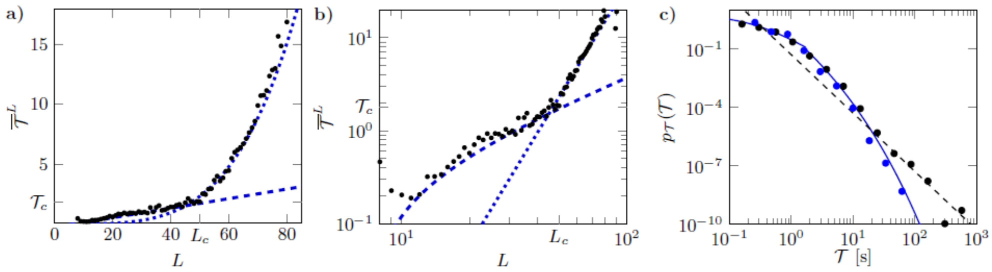

Supplementary Figure S4: Characteristics and predictive power of the mean field model. Average travel time $\overline{\mathcal{T}}^L$ as a function of trajectory length L in **a)** linear scale **b)** loglog scale (same as inset of Fig. 2**c** in the main manuscript). The linear and power law tendencies (Eq. (S10)) are shown respectively as dashed and dotted blue lines. The transition travel time and trajectory lengths between these two tendencies are indicated as $\mathcal{T}_c$ and L_c. **c)** Full travel time PDF (black dots) compared to the numerical mean field travel time PDF (blue dots). The theoretical mean field model is shown as a blue line. The late time power law tendency $p_{\mathcal{T}} \sim \mathcal{T}^{-3}$ characteristic of noise-driven anomalous transport in the late time regime is shown as a black dashed line.

E. Noise in vessel transit time induced by random network fluctuations

The series of local transit times along a given trajectory exhibit large erratic fluctuations around the series of average transit times (Supplementary Figure S5a). To analyze these fluctuations, induced by the randomness of the network, we define a noise term ξ by normalizing the vessel transit time t_j at the jth step by its mean value $\overline{t_j}^L$, where $\overline{\cdot}^L$ denotes the average over trajectories of size L,

$$\xi = \frac{t_j}{\overline{t_j}^L}. \tag{S13}$$

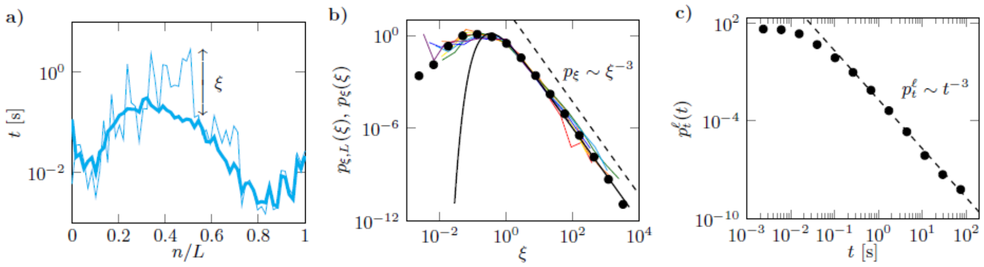

Supplementary Figure S5: Statistics of Lagrangian capillary transit times. a) Series of local and averaged transit times for trajectories of equal lengths ($L = 60$), as a function of the number n of vessels visited since the inlet arteriole. The thick line shows the average trend (as in the inset of Fig. 2**b** in the main manuscript) and the thin line shows an example of transit time series along one trajectory. The noise ξ is defined as the ratio of the local to the average transit time at a given position n/L (Eq. (S13)). **b)** Noise probability density functions. Colored lines show the noise PDF $p_{\xi,L}(\xi)$ for different trajectory lengths $L = \{20, 30, 40, 50, 60, 70, 80\}$ with the color convention from Fig. 2 of the main manuscript. The black dots show the noise PDF averaged over all trajectory lengths $p_\xi(\xi)$. The black line represents the analytical approximation of Eq. (S15) for large noise values. The dashed line shows the power law trend for large fluctuations. **c)** Lagrangian transit time PDFs (black dots). The dashed line shows the power law trend (Eq. (S14)).

The statistics of these fluctuations p_ξ are found to be independent on the trajectory length (Supplementary Figure S5b). Large fluctuations of ξ, which control long times, follow a power law $p_\xi(\xi) \sim \xi^{-3}$. This trend is consistent with the Lagrangian transit times distribution, which follows the same power law scaling (Supplementary Figure S5c). This power law behavior is linked to the Eulerian vessel transit time distribution, which follows $p_t^e(t) \sim t^{-2}$ at large times (Eq. 3 in the main manuscript). Indeed, the Lagrangian transit times statistics $p_t^\ell(t)$, that is the statistics of vessel transit times sampled by particle trajectories, are weighted by the local flow rate $Q \sim 1/t$ [17], leading to

$$p_t^\ell(t) \sim p_t^e(t)/t \sim t^{-3}. \tag{S14}$$

As discussed in the main manuscript, this power law scaling for long transit times is induced by the uniform distribution of low flow rates (see Fig. 1**b** in the main manuscript). Such uniform distributions are characteristic of flow in random networks (Supplementary FigureS3**d**), which follow exponential distributions and therefore are constant at low values [15]. Assuming an exponential model of flow rate fluctuations induced by random network connections, we obtain, after change of variable and weighting by flow, the following noise PDF for fluctuations of vessel

transit times,

$$p_\xi(\xi) = \frac{\exp(-1/\xi)}{\xi^3}, \tag{S15}$$

which is in good agreement with measured noise in the large fluctuation range, i.e. for $\xi \geq 1$ (Supplementary Figure S5b).

F. Analytical derivation of the Continuous Time Random Walk model

In complement to the mean field transport model that captures the effect of dipole-driven trajectory lengths distributions, we derived a stochastic transport model integrating in addition the effect of network-driven transit time fluctuations. Fluid elements move to one vessel to the next with a broadly varying transit time, which corresponds closely to the Continuous Time Random Walk representation [18–20]. In this framework, the travel time $\mathcal{T}_j$ after $j-1$ vessels evolves as:

$$\mathcal{T}_{j+1} = \mathcal{T}_j + t_j \tag{S16}$$

with t_j the time increments at vessel j. From Eq. (S13) we take $t_j = \xi \overline{t_j}^L$, where ξ_j is a random noise with distribution $p_\xi(\xi)$. From the mean field model (Eq. (S10)) we have $\overline{t_j}^L = \tau_1$ for $L < L_c$ and $\overline{t_j}^L = \frac{\mathcal{T}_c(L/L_c)^4 - \mathcal{T}_c}{L-L_c}$ for $L > L_c$, leading to,

$$\begin{cases} t_j = \xi_j \tau_1 & L_0 \leq j < L_c \\ t_j = \xi_j \dfrac{\mathcal{T}_c(L/L_c)^4 - \mathcal{T}_c}{L - L_c} & L_c \leq j < L \end{cases} \tag{S17}$$

The temporal increment in the CTRW model (Eq. (S16)) hence integrates the mean field field travel time distribution driven by the dipole flow patterns through $\overline{t_j}^L$ and the network fluctuations quantified by the noise term ξ_j. Because we focus on the impact of long travel times, we do not consider trajectories smaller than L_0, which is the smallest trajectory length in the mean field model (Supplementary Figure S4**a**). Therefore, the CTRW model is initialized at $\mathcal{T}_{L_0} = \mathcal{T}_0$.

The total network travel time is,

$$\mathcal{T} = \mathcal{T}_0 + \sum_{j=L_0}^{L-1} t_j \tag{S18}$$

Since the time increments t_j are independent, the travel time PDF is,

$$p_\mathcal{T}(\mathcal{T}) = \sum_{L=L_0+1}^{\infty} p_L(L) \int_0^\infty dt_{L_0} ... \int_0^\infty dt_{L-1} p_{t,L_0}(t_{L_0}) ... p_{t,L-1}(t_{L-1}) \delta \left(\mathcal{T}_0 + \sum_{j=L_0}^{L-1} t_j - \mathcal{T} \right) \tag{S19}$$

Since the trajectory distribution $p_L(L)$ and the relation between mean travel time and length follow different regimes above and below L_c, we decompose Eq. (S19) as a sum of two contributions:

$$p_\mathcal{T}(\mathcal{T}) = p^1_\mathcal{T}(\mathcal{T}) + p^2_\mathcal{T}(\mathcal{T}) \tag{S20}$$

where

$$p^1_{\mathcal{T}}(\mathcal{T}) = \sum_{L=L_0+1}^{L_c} p_L(L) \int_0^\infty d\xi_{L_0} ... \int_0^\infty d\xi_{L-1} p(\xi_{L_0})...p(\xi_{L-1}) \delta \left(\mathcal{T}_0 + \tau_1 \sum_{j=L_0}^{L-1} \xi_j - \mathcal{T} \right), \quad \text{(S21)}$$

$$p^2_{\mathcal{T}}(\mathcal{T}) = \sum_{L=L_c+1}^{\infty} p_L(L) \int_0^\infty d\xi_{L_0} ... \int_0^\infty d\xi_{L-1} p(\xi_{L_0})...p(\xi_{L-1})$$
$$\delta \left(\mathcal{T}_0 + \tau_1 \sum_{j=L_0}^{L_c-1} \xi_j + \frac{\mathcal{T}_c (L/L_c)^4 - \mathcal{T}_c}{L - L_c} \sum_{j=L_c}^{L-1} \xi_j - \mathcal{T} \right). \quad \text{(S22)}$$

Eq. (S21) and Eq. (S22) can be expressed in Laplace space respectively as,

$$\tilde{p}^1_{\mathcal{T}}(s) = e^{-s\mathcal{T}_0} \sum_{L=L_0+1}^{L_c} p_L(L) \mathcal{P}^{L-L_0}(s\tau_1) \quad \text{(S23)}$$

and

$$\tilde{p}^2_{\mathcal{T}}(s) = e^{-s\mathcal{T}_0} \mathcal{P}^{L_c-L_0}(s\tau_1) \sum_{L=L_c+1}^{\infty} p_L(L) \mathcal{P}^{L-L_c} \left(s \frac{\mathcal{T}_c (L/L_c)^4 - \mathcal{T}_c}{L - L_c} \right) \quad \text{(S24)}$$

where $\mathcal{P} = \mathcal{L}\{p_\xi(\xi)\}$ is the Laplace transform of $p_\xi(\xi)$. For the noise PDF $p_\xi(\xi)$ we take the analytical expression of Eq. (S15), which provides a good approximation for large fluctuations $\xi \geq 1$ (Supplementary Figure S5). Its Laplace transform is $\mathcal{P}(s) = 2sK_2(2\sqrt{s})$ with s the Laplace variable and K_2 is a modified Bessel function of the second kind.

The CTRW model (Eq. (S20)), solved by numerical Laplace inversion of Eq. (S23) and Eq. (S24), provides an accurate prediction of the advective travel time PDF for $\mathcal{T} > \mathcal{T}_0$ (Fig. 4**a** in the main manuscript) with no fitting parameter. This model is thus fully determined from the trajectory length PDF (Eq. (S11)) and the relationship between average time and trajectory length (Eq. (S10)). Model predictions are also consistent with the travel time distributions of oxygen and amyloid-β up to a cutt off time driven by diffusion.

G. Reference CTH model

To investigate the effect of the long network travel times, which cannot be accessed by in vivo measurements, we compare our model prediction to that of a reference CTH model calibrated from experimental data [21]. The latter assumes that the travel time PDF follows a Gamma distribution,

$$p_{\mathcal{T}}(\mathcal{T}) = \frac{\mathcal{T}^{n-1}}{\Gamma(n)\theta^n} e^{-\mathcal{T}/\theta} \quad \text{(S25)}$$

where the parameters n and θ are related to the travel time mean and variance as $\overline{\mathcal{T}} = n\theta$ and $\sigma^2_{\mathcal{T}} = n\theta^2$, respectively. Jespersen et al. [21] have compiled a series of experimental data obtained in various physiological conditions and shown that these moments follow the approximate relationship $\sigma_{\mathcal{T}} \approx 0.7\overline{\mathcal{T}}$ (see symbols in Figure 4 of Jespersen et al. [21]). Using this relationship and the average travel time in our simulations, $\overline{\mathcal{T}} = 0.66$ s, we estimate the parameters of the reference model to be $n = 2$ and $\theta = 0.32$ for the considered microvascular network. Since experimentally measured travel time distributions are limited to times smaller than ~ 5 s due to blood recirculation [22,23], this empirical model serves here as a reference to assess the effect of neglecting the experimentally inaccessible longest travel times.

H. Robustness of flow and transport properties across anatomical networks, impact of stalling and diameter adaptations

We have previously shown [13] that the size of a representative elementary volume (REV) for the capillary bed is ($\sim$400x400x400 μm^3). Thus, the microvessel network (Supplementary Figure S1.a) used in the present simulations contains more than 10 representative elementary volumes (REV) for the capillary bed. Moreover, for this network, the ratio between the number of penetrating arterioles and of ascending venules, which gives insight on the large-scale structures superimposed to the capillary bed, corresponds to the average ratio in the cortex. Thus, we expect to obtain statistically robust results.

In this section, we check this and further test the generality of our results using two different micro-vascular networks: the same network as shown in Supplementary Figure S1.a, but with 10 % randomly stalled capillaries, and another network extracted from the same region of the mouse brain previously obtained by Tsai et al. [1] and Blinder et al. [2]. The flow statistics are found to be very similar in all networks and consistent with our stochastic model (Supplementary Figure S6.a). As a consequence, our transport model is in good agreement with the travel time distributions in all networks (Supplementary Figure S6.b).

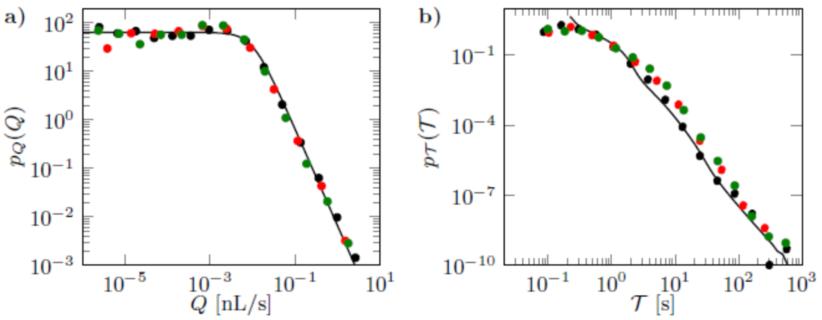

Supplementary Figure S6: Statistics of flow and transport in a stalled micro-vascular network and in a different sample network. a) Flow rate PDFs. **b)** Travel time PDFs. Black dots represent the results from the network presented in Supplementary Figure S1. Red dots represent the results for this same network with 10% of stalled capillaries. Green dots represent the results for another network drawn from the same region of the mouse brain. The continuous lines in sub-figure a) and b) represent respectively our flow PDF (Eq. (1) in the main text) and transport models (Eq. (7) in the main text).

Moreover, we evaluated the impact of diameter variations on the flow and transport statistics. These temporal fluctuations can be induced by passive or active mechanisms related to changes in pressure, neurovascular coupling and/or cerebral autoregulation [24]. Long term vascular remodeling may also occur in disease [25]. The associated vessel diameter variations, typically in the range -30 to 20%, are much smaller than the diameter variations encountered from vessel to vessel in the anatomical network under study. Thus, to assess an upper bound of their impact on our results, we computed the flow and transport statistics in the same anatomic network, where we imposed a uniform distribution of conductances. Such modification is more drastic than if induced by any conceivable physiological or pathological mechanism leading to vessel diameter variations. Yet, it only results in slight corrections of the flow and transport properties, mainly in the short transit time and intermediate travel time regimes, as displayed in Supplementary Figure S7. In the same way, we have verified that neglecting the complex rheology of blood leads

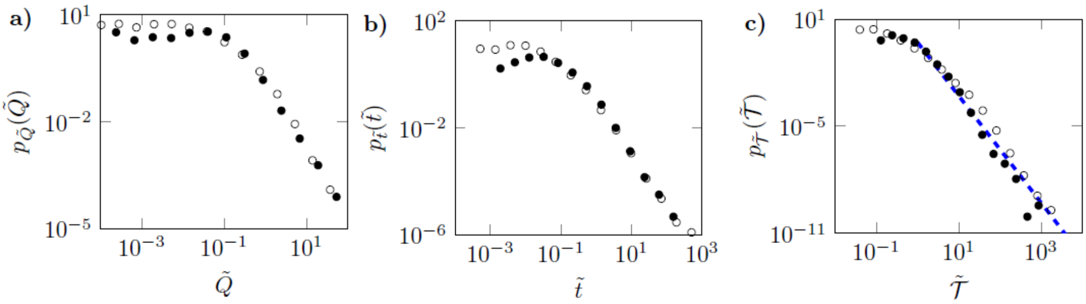

Supplementary Figure S7: Comparison of flow and transport statistics simulated on the anatomical network with heterogeneous and homogeneous conductances. a) Flow rate PDF. **b)** Vessel transit time PDF. **c)** Network travel time PDF. **a-c)** All PDFs are computed for the anatomical network displayed in Supplementary Figure S1**a-b** (dots), and for the same microvascular network in which all conductances have been modified to be uniform (circles). They are normalized by their average value.

to even smaller impact. In both cases, with regard to the travel time distribution (Supplementary Figure S7.b), the late time regime is unaffected, and the power law with exponent -3 is still valid.

I. Reactive transport of oxygen

To investigate the impact of the travel time distribution on oxygen transport within the brain cortex, we use a simplified Lagrangian model of transport in vessels coupled to diffusive mass transfer and consumption in the brain tissue. We consider a transported element of fluid carrying an oxygen concentration c_{O_2}. Diffusive mass transfer from the vessel to the tissue is modeled by a first-order exchange kinetics [26, 27],

$$\frac{\partial c_{O_2}}{\partial t} = -k(c_{O_2} - c^T_{O_2}) \tag{S26}$$

where $c^T_{O_2}$ is the local averaged concentration in the brain tissue next to the vessel and k^{-1} is the characteristic diffusion time across the vessel walls. The oxygen concentration in the tissue $c^T_{O_2}$ evolves through consumption and diffusive mass transfer with the vessel as,

$$\frac{\partial c^T_{O_2}}{\partial t} = -k_{O_2} c^T_{O_2} + k(c_{O_2} - c^T_{O_2}) \tag{S27}$$

where k_{O_2} is the first order kinetic constant for oxygen consumption in the brain tissue, assumed here to be uniform accros the depth of the cortex. Since the characteristic diffusion time across the vessel walls, $\frac{e^2}{D_{O_2}} \sim 5 \times 10^{-4} s$ (taking the wall thickness $e \sim 1\mu$m for capillary vessels [28]), is small, we assume that $k >> k_{O_2}$, so that oxygen in the tissue is locally in equilibrium with oxygen in blood ($c^T_{O_2} \simeq c_{O_2}$). This yields:

$$\frac{\partial c_{O_2}}{\partial t} = -k_{O_2} c_{O_2}, \tag{S28}$$

a first order equation similar as the one used by [21]. Solving for this equation yields:

$$c_{O_2}(t) = c_{O_2}(0) \exp\left(-k_{O_2} t\right) \tag{S29}$$

where t is the local travel time from the inlet along a given trajectory and $c_{O_2}(0) = c^A_{O_2}$ is the oxygen concentration at the arterial inlets. Because the kinetics are linear, the oxygen concentration carried by each fluid element can be resolved independently and then added to reconstruct the oxygen distribution in the network. In other words, the average concentration in a given vessel is deduced as the average of local concentrations of all trajectories flowing through this vessel, yielding the oxygen concentration field throughout the network for a given kinetic constant k_{O_2} (Fig. 5**a,b** in the main manuscript).

J. Amyloid-β clearance

The transport mechanisms of amyloid-β are complex and still poorly understood. They involve LRP1 dependent transport, reaction of amyloid-β with soluble factors (apoJ, apoE, sLRP1) as well as at least four endocytotic/transcytotic systems [29]. However, experimental studies using radio-labeled or other tracers to study the clearance of amyloid-β from brain to blood have consistently shown an exponential decrease of the tracer concentration with time, in mice models of Alzheimer's (e.g. [30, 31]) as well as in humans (e.g. [32, 33]), i.e. at the scale of the brain, suggesting that first-order kinetics is a good approximation for this process. Similar results have been obtained at the local scale with an in vitro blood-brain barrier model based on hCMEC/D3 endothelial monolayers [31]. Hence, first-order kinetics descriptions of amyloid-β transport are commonly used in pharmacokinetic models (e.g. [34]). Here we follow this approach and assume a linear mass transfer between the tissue and the vessels.

Furthermore, due to tissue production, the tissue amyloid-β concentration, $c^T_{A_\beta}$, is much higher than the blood amyloid-β concentration, $c^T_{A_\beta}$ (see e.g. [35] where $c^A_{A_\beta}$=264 pg/ml and [34] where $c^T_{A_\beta}$=8150 pg/ml, as measured by the same immunoprecipitation/mass spectrometry technique). Thus, we assume that $c^T_{A_\beta}$ is approximately constant because of tissue production. Therefore, we obtain,

$$\frac{d(c_{A_\beta})}{dt} = \frac{d(c_{A_\beta} - c^T_{A_\beta})}{dt} = -k_{A_\beta}(c_{A_\beta} - c^T_{A_\beta}) \tag{S30}$$

where $k^{-1}_{A_\beta}$ is the characteristic time for amyloid-β clearance. Solving this equation leads to:

$$c_{A_\beta}(t) - c^T_{A_\beta} = (c_{A_\beta}(0) - c^T_{A_\beta}) \exp\left(-k_{A_\beta} t\right) \tag{S31}$$

where $c_{A_\beta}(0) = c^A_{A_\beta}$ is the amyloid-β concentration at the arterial inlets.

Thus, for each trajectory with travel time $\mathcal{T}$, the ratio between the venous outlet concentration $c^v_{A\beta}$ and tissue concentration is given by,

$$c^v_{A\beta}/c^T_{A\beta} = 1 - (1 - \frac{c^A_{A\beta}}{c^T_{A\beta}}) \exp\left(-k_{A\beta}\mathcal{T}\right) \tag{S32}$$

This simplifies to

$$c^v_{A\beta}/c^T_{A\beta} = 1 - \exp\left(-k_{A\beta}\mathcal{T}\right) \tag{S33}$$

when the travel time is much larger than $\frac{c^A_{A\beta}}{c^T_{A\beta}} k^{-1}_{A_\beta}$. As for the oxygen model, we couple this first order model of amyloid production in tissues with the typical measured arterio-venous increase of 20% [36], and obtain $\tau_r^{A_\beta} = k^{-1}_{A_\beta} = 97$ s. Inserting the critical times $\tau_c^{A\beta} = 8, 16$ or 40 s in Eq. (S32) yields respectively a three-, five- or tenfold arterio-venous increase of the total intravascular amyloid concentration.

References

[1] Tsai, P. S. *et al.* Correlations of neuronal and microvascular densities in murine cortex revealed by direct counting and colocalization of nuclei and vessels. *J. Neurosci.* **29** 14553–14570 (2009).

[2] Blinder, P. *et al.* The cortical angiome: an interconnected vascular network with noncolumnar patterns of blood flow. *Nat. Neurosci.* **16**, 889–897 (2013).

[3] Pries, A. R., Secomb, T. W., Gaehtgens, P., & Gross,J. Blood flow in microvascular networks. Experiments and simulation. *Circ. Res.*, **67**, 826–834 (1990).

[4] Lorthois, S., Cassot, F. & Lauwers, F. Simulation study of brain blood flow regulation by intra-cortical arterioles in an anatomically accurate large human vascular network. Part II: Flow variations induced by global or localized modifications of arteriolar diameters. *NeuroImage* **54**, 2840–2853 (2011).

[5] Cruz-Hernández, J. C. *et al.* Neutrophil adhesion in brain capillaries reduces cortical blood flow and impairs memory function in Alzheimer's disease mouse models. *Nat. Neurosci.* **22**, 413–420 (2019).

[6] Peyrounette, M., Davit, Y., Quintard, M. & Lorthois, S. Multiscale modelling of blood flow in cerebral microcirculation: Details at capillary scale control accuracy at the level of the cortex. *PLoS One* **13**, e0189474 (2018).

[7] Pries, A. R. *et al.* Resistance to blood flow in microvessels in vivo. *Circ. Res.* **75**, 904-915 (1994).

[8] Lorthois, S. Blood suspension in a network. In Dynamics of Blood Cell Suspensions in Microflows. CRC Press, 257-286 (2019).

[9] Pries, A. R., Reglin, B. & Secomb, T. W. Structural response of microcirculatory networks to changes in demand: information transfer by shear stress. *Am. J. Physiol. Heart Circ.* **284**, H2204-H2212 (2003).

[10] Fry, B. C., Lee, J., Smith, N. P. & Secomb, T. W. Estimation of blood flow rates in large microvascular networks. *Microcirculation* **19**, 530-538 (2012).

[11] Holter, K. E. *et al.* Interstitial solute transport in 3D reconstructed neuropil occurs by diffusion rather than bulk flow. *Proc. Natl. Acad. Sci.* **114**, 9894-9899 (2017).

[12] Kurowski, P., Ippolito, I., Hulin, J., Koplik, J. & Hinch, E. Anomalous dispersion in a dipole flow geometry. *Phys. Fluids* **6**, 108–117 (1994).

[13] Smith, A. F. *et al.* Brain capillary networks across species: a few simple organizational requirements are sufficient to reproduce both structure and function. *Front. Physiol.* **10**, 233 (2019).

[14] Duvernoy, H. M., Delon, S. & Vannson, J. Cortical blood vessels of the human brain. *Brain Res. Bull.* **7**, 519–579 (1981).

[15] Alim, K., Parsa, S., Weitz, D. A. & Brenner, M. P. Local pore size correlations determine flow distributions in porous media. *Phys. Rev. Lett.* **119**, 144501 (2017).

[16] Schmid, F., Tsai, P. S., Kleinfeld, D., Jenny, P. & Weber, B. Depth dependent flow and pressure characteristics in cortical microvascular networks. *PLoS Comput. Biol.* **13**, e1005392 (2017).

[17] Dentz, M., Kang, P. K., Comolli, A., Le Borgne, T. & Lester, D. R. Continuous time random walks for the evolution of lagrangian velocities. *Phys. Rev. Fluid.* **1**, 074004 (2016).

[18] Kang, P. K., Dentz, M., Le Borgne, T. & Juanes, R. Spatial Markov model of anomalous transport through random lattice networks. *Phys. Rev. Lett.* **107**, 180602 (2011).

[19] Metzler, R. & Klafter, J. The random walk's guide to anomalous diffusion: a fractional dynamics approach. *Phys. Rep.* **339**, 1–77 (2000).

[20] De Anna, P. *et al.* Flow intermittency, dispersion, and correlated continuous time random walks in porous media. *Phys. Rev. Lett.* **110**, 184502 (2013).

[21] Jespersen, S. N. & Østergaard, L. The roles of cerebral blood flow, capillary transit time heterogeneity, and oxygen tension in brain oxygenation and metabolism. *J. Cereb. Blood Flow Metab.* **32**, 264–277 (2012).

[22] Gutierrez-Jimenez, E. *et al.* Effect of electrical forepaw stimulation on capillary transit-time heterogeneity (CTH). *J. Cereb. Blood Flow Metab.* **36**, 2072–2086 (2016).

[23] Merkle, C. W. & Srinivasan, V. J. Laminar microvascular transit time distribution in the mouse somatosensory cortex revealed by Dynamic Contrast Optical Coherence Tomography. *NeuroImage* **125**, 350–362 (2016).

[24] Fry, B. C., Roy, T. K. & Secomb, T. W. Capillary recruitment in a theoretical model for blood flow regulation in heterogeneous microvessel networks. *Physiol. Rep.* **1** (2013).

[25] Nortley, R. *et al.* Amyloid beta oligomers constrict human capillaries in Alzheimer's disease via signaling to pericytes. *Science* **365** (2019).

[26] Gagnon, L. *et al.* Modeling of cerebral oxygen transport based on in vivo microscopic imaging of microvascular network structure, blood flow, and oxygenation. *Front. Comput. Neurosci.* **10** (2016).

[27] Berg, M., Davit, Y., Quintard, M. & Lorthois, S. Modelling solute transport in the brain microcirculation: is it really well mixed inside the blood vessels? *J. Fluid Mech.* **884** (2020).

[28] Kutuzov, N., Flyvbjerg, H. & Lauritzen, M. Contributions of the glycocalyx, endothelium, and extravascular compartment to the blood–brain barrier. *Proc. Natl. Acad. Sci.* **115**, E9429-E9438 (2018).

[29] Hladky, S. B. & Barrand, M. A. Elimination of substances from the brain parenchyma: efflux via perivascular pathways and via the blood–brain barrier. *Fluids Barriers CNS* **15**, 1-73 (2018).

[30] Shibata, M. *et al.* Clearance of Alzheimer's amyloid beta1-40 peptide from brain by LDL receptor related protein 1 at the blood-brain barrier. *J. Clin. Investig.* **106** 1489–1499 (2000).

[31] Swaminathan, S. K. *et al.* Insulin differentially affects the distribution kinetics of amyloid beta 40 and 42 in plasma and brain. *J. Cereb. Blood Flow Metab.* **38**, 904–918 (2018).

[32] Bateman, R. J. *et al.* Human amyloid-β synthesis and clearance rates as measured in cerebrospinal fluid in vivo. *Nat. Med.* **12**, 856-861 (2006).

[33] Mawuenyega, K. G. *et al.* Decreased clearance of CNS β-amyloid in Alzheimer's disease. *Science* **330**, 1774-1774 (2010).

[34] Potter, R. *et al.* Increased in vivo amyloid 42 production, exchange, and loss in presenilin mutation carriers. *Sci. Transl. Med.* **5**, 189ra77–189ra77 (2013).

[35] Roberts, K. F. *et al.* Amyloid efflux from the central nervous system into the plasma: Brain efflux of amyloid. *Ann. Neurol.* **76**, 837–844 (2014).

[36] Xiang, Y. *et al.* Physiological amyloid beta clearance in the periphery and its therapeutic potential for Alzheimer's disease. *Acta Neuropathol.* **130**, 487–499 (2015).
