# Supplementary material for: Network-driven anomalous transport is a fundamental component of brain microvascular dysfunction
Source: Nat Commun. 2021 Dec 15;12:7295. doi: 10.1038/s41467-021-27534-8 (PMC8674232; doi:10.1038/s41467-021-27534-8)
Supplement: Supplementary file 4 — Description of Additional Supplementary Files [file 41467_2021_27534_MOESM4_ESM.pdf]

Title: Supplementary Movie 1 :
Description: Supplementary Movie 1 shows a rotating view of the first 1mm3 mouse brain sample used to model the brain blood flow transport properties, corresponding to the snapshots of Supplementary Figure 1a-b. Arterioles are displayed in red, venules in blue and capillary vessels in green.

Title: Supplementary Movie 2 :
Description: Supplementary Movie 2 shows a rotating view of the flow rate distribution, highlighting the two flow regimes, corresponding to the snapshots of Fig. 1a and Supplementary Figure 1f. Vessel flow rates are represented with blue shades for QQc.

Title: Supplementary Movie 3 :
Description: Supplementary Movie 3 shows a rotating view of all trajectories with $L \leq 30$ (orange) and $L \geq 60$ (blue) originating from the arteriole highlighted by an arrow in Fig. 1c, corresponding to the snapshot of Fig. 1c.

Title: Supplementary Movie 4 :
Description: Supplementary Movie 4 shows a rotating view of typical particle trajectories with different trajectory lengths (numbers of visited vessels)(Red: L=20; Yellow: L=30; Orange: L=40; Green: L=50; Cyan: L=60; Blue: L=70; Violet: L=80) corresponding to the snapshot of Fig. 2a.

Title: Supplementary Movie 5 :
Description: Supplementary Movie 5 shows a rotating view of the oxygen concentration field, corresponding to the snapshots of Fig. 5a-b.
